# Current status and future perspectives of fractional flow reserve derived from invasive coronary angiography

**DOI:** 10.3389/fcvm.2023.1181803

**Published:** 2023-06-06

**Authors:** Milan Dobrić, Matija Furtula, Milorad Tešić, Stefan Timčić, Dušan Borzanović, Nikola Lazarević, Mirko Lipovac, Mihajlo Farkić, Ivan Ilić, Darko Boljević, Jelena Rakočević, Srđan Aleksandrić, Stefan Juričić, Miodrag Ostojić, Milovan Bojić

**Affiliations:** ^1^ Cardiology Clinic, Institute for Cardiovascular Diseases “Dedinje”, Belgrade, Serbia; ^2^ University of Belgrade Faculty of Medicine, Belgrade, Serbia; ^3^ Cardiology Clinic, University Clinical Centre of Serbia, Belgrade, Serbia; ^4^ Institute of Histology and Embryology “Aleksandar Đ. Kostić”, Faculty of Medicine, University of Belgrade, Belgrade, Serbia

**Keywords:** fractional flow reserve, virtual FFR, 3D reconstruction, computational flow dynamic (CFD), coronary angiography

## Abstract

Assessment of the functional significance of coronary artery stenosis using invasive measurement of fractional flow reserve (FFR) or non-hyperemic indices has been shown to be safe and effective in making clinical decisions on whether to perform percutaneous coronary intervention (PCI). Despite strong evidence from clinical trials, utilization of these techniques is still relatively low worldwide. This may be to some extent attributed to factors that are inherent to invasive measurements like prolongation of the procedure, side effects of drugs that induce hyperemia, additional steps that the operator should perform, the possibility to damage the vessel with the wire, and additional costs. During the last few years, there was a growing interest in the non-invasive assessment of coronary artery lesions, which may provide interventionalist with important physiological information regarding lesion severity and overcome some of the limitations. Several dedicated software solutions are available on the market that could provide an estimation of FFR using 3D reconstruction of the interrogated vessel derived from two separated angiographic projections taken during diagnostic coronary angiography. Furthermore, some of them use data about aortic pressure and frame count to more accurately calculate pressure drop (and FFR). The ideal non-invasive system should be integrated into the workflow of the cath lab and performed online (during the diagnostic procedure), thereby not prolonging procedural time significantly, and giving the operator additional information like vessel size, lesion length, and possible post-PCI FFR value. Following the development of these technologies, they were all evaluated in clinical trials where good correlation and agreement with invasive FFR (considered the gold standard) were demonstrated. Currently, only one trial (FAVOR III China) with clinical outcomes was completed and demonstrated that QFR-guided PCI may provide better results at 1-year follow-up as compared to the angiography-guided approach. We are awaiting the results of a few other trials with clinical outcomes that test the performance of these indices in guiding PCI against either FFR or angiography-based approach, in various clinical settings. Herein we will present an overview of the currently available data, a critical review of the major clinical trials, and further directions of development for the five most widely available non-invasive indices: QFR, vFFR, FFRangio, caFFR, and AccuFFRangio.

## Introduction

Functional evaluation of epicardial coronary stenosis remains a very frequent scenario in interventional practice. During the last few decades, many invasive indices were proposed and validated, while few of them are integrated into contemporary clinical guidelines. Despite having been proven to improve outcomes, invasive functional evaluation remains underutilized worldwide. Some of the factors that influence this are the additional price of the wires with pressure sensors, prolongation of the procedure due to the conduction of very precise steps of the measurement, induction of hyperemia (for hyperemic indices like FFR), possibility to cause complication with the instrumentalization of the coronary artery, and sometimes the change of the geometry of the artery with wire introduction.

The ideal parameter would be the one that does not prolong the procedure significantly, is performed online (in the catheterization laboratory), uses standard diagnostic angiograms, and gives the operator clear information on coronary physiology superimposed on the angiographic picture.

In recent years, many novel functional indices derived from standard coronary angiography were proposed. The first step for all these methods is the creation of the three-dimensional model of the coronary artery (3D Quantitative Coronary Angiography—3D QCA), using two separate two-dimensional angiographic projections. For such a 3D model, a computer simulation of flow is performed. The best estimation of blood flow in such a tube could be made using Computational flow dynamics (CFD) and Navier-Stokes equations, but this could be time-consuming and require extensive computational power, limiting the ability of such a system to be used online during the procedure.

Consequently, this step could be simplified using different computational approaches and approximations that could speed up the whole process and provide the result in just a few minutes. Some of these methods use only patient-specific anatomical features, while others use some of the physiological inputs as well (e.g., aortic pressure, TIMI frame count). Boundary conditions (especially for the distal end of the epicardial vessel) that are needed for these calculations are estimated or used as population averages. The output is usually a color-numeric scheme superimposed on the vessel geometry, giving the operator information on pressure drop and calculated FFR in different vessel segments. Furthermore, additional QCA data are also available, like diameters, lengths, diameter stenosis, etc. Recently, a novel reduced-order modeling (ROM method) of blood flow in coronary arteries was shown to be able to replace complex and time-consuming CFD-based FFR estimation and provide real-time results (1). With this innovative approach calculations could be 25 times faster than using conventional full-order model, while maintaining acceptable prediction errors (2).

Since this is an area of intensive investigation, and these systems are constantly evolving, interventionalists need to know the basic technical characteristics, similarities, and differences between different technologies, as well as their diagnostic performance and PCI outcomes. The aim of this article is to summarize available information for the five most widely available angiography-based FFR solutions: QFR, vFR, FFRangio, caFFR, and AccuFFRAngio. Table 1 summarizes the most important characteristics of these parameters.

**Table 1:** Overview of the five most widely available angiography derived FFR parameters.

Index	Company name	Anatomic region	Number of angiographic projections needed	Aortic pressure measurements	TIMI frame count	Minimum frames per second (fps)	Clinical studies comparing with FFR	Studies with clinical outcomes of PCI guided by the index
QFR	Medis Medical Imaging, Leiden, the Netherlands	Single vessel	2 projections with minimal separation of 25°	NO	YES	15 (or even 7.5) (3)	FAVOR Pilot (2016) (4) FAVOR II China (2017) (5) FAVOR II E-J (2018) (6) WIFI II (2018) (7) Choi et al. (2021) (8)	FAVOR III China (2021) (9) FAVOR III E-J * (NCT03729739) FAVOR4-QVAS * (NCT03977129) PIONEER IV * (NCT04923191)
vFFR	Pie Medical Imaging, Maastricht, the Netherlands	Single vessel	2 projections with minimal separation of 30°	YES	NO	7.5	FAST (2020) (10) FAST EXTEND (2021) (11) FAST II (2022) (12)	FAST III * (NCT04931771) LIPSIA STRATEGY * (NCT03497637)
FFRangio	CathWorks, Kfar Saba, Israel	Multi-vessel (3D coronary tree)	2 or 3 projections with minimal separation of 30°	NO	NO	10	Kornowski et al. (2016) (13) Pellicano et al. (2017) (14) Kornowski et al. (2018) (15) FAST-FFR (2019) (16) Omori et al. (2019) (17) Witberg et al. (2020) (18)	Witberg et al. (2022) (19) Japan FFRangio Clinical Outcomes Study * (NCT05648396)
caFFR	Rainmed, China	Single vessel	2 projections with minimal separation of 30°	YES, simultaneous recording of aortic pressure by specialized pressure transducer	YES	15	FLASH FFR (2020) (20) Ai et al. (2021) (21)	FLASH II * (NCT04575207)
AccuFFRangio	ArteryFlow Technology, Hangzhou, China	Single vessel	2 projections with minimal separation of 25°	YES	YES	15	Li et al. (2021) (22) Jiang et al. (2022) (23)	AccuFFRangio in STEMI (NCT05209503) AccuFFRangio in NSTEMI (NCT05202041)

* Ongoing or planned trials.

## Diagnostic performance of angiography-based FFR indices

First attempts to estimate the functional significance of coronary artery stenoses using 3-dimensional (3D) quantitative coronary angiography (QCA) and TIMI (Thrombolysis In Myocardial Infarction) frame count for fast computation of FFR in patients with coronary artery disease showed good correlation with wire based FFR (*r* = 0.81, *p* < 0.001), and the area under the receiver-operating characteristic (AUC) of 0.93 (24). Since then, many novel functional parameters based on the 3D reconstruction of the interrogated vessel were proposed (4, 10, 13, 20, 22). They all were initially evaluated and compared to FFR as the gold standard, proving their good correlation and diagnostic accuracy. On the other hand, the amount of data on PCI guidance by these indices with clinical outcomes is limited, and few trials are still recruiting participants.

### Quantitative flow ratio (QFR)

FAVOR Pilot study (*Functional Assessment by Various Flow Reconstructions*) was the first trial to compare QFR (*Medis Medical Imaging System, Leiden, the Netherlands*) with wire-based FFR (4). For the purpose of this trial, three QFR flow models were proposed and compared to FFR: (1) fQFR (using a fixed empiric hyperemic coronary flow velocity of 0.35 m/s that was measured in previous studies for calculation), (2) cQFR (frame count analysis was used separately on the 2 diagnostic angiographic projections, with no pharmacologically induced hyperemia), and (3) aQFR (using frame count analysis separately on the 2 angiographic projections acquired during hyperemia). A comparison of all three QFR values to FFR was performed at the position that corresponded to the position of the sensor of the pressure wire used for FFR measurement. A cut-off value for all QFR models was set to 0.80, just as it was with wire-based FFR. QFR values were computed *post hoc* in a core-lab setting, not during the invasive procedure. This trial included 84 coronary vessels in 73 patients and showed good agreement of fQFR, cQFR, and aQFR parameters with FFR, with mean differences of 0.003 ± 0.068 (*p* = 0.66), 0.001 ± 0.059 (*p* = 0.90), and −0.001 ± 0.065 (*p* = 0.90), respectively. Diagnostic accuracy for detecting an FFR value of 0.80 or less was 80% (fQFR), 86% (cQFR), and 87% (aQFR). Most importantly, it was shown that the cQFR model, which does not require induction of hyperemia, showed a higher AUC than fQFR (difference: 0.04; 95% CI: 0.01 to 0.08; *p* < 0.01), and showed no significant difference between aQFR (difference: 0.01; 95% CI: −0.04 to 0.06; *p* = 0.65). This supports the use of the QFR model without hyperemia for fast, simple, and accurate determining of functional significance of the lesions.

The FAVOR II China study (*Functional Diagnostic Accuracy of Quantitative Flow Ratio in Online Assessment of Coronary Stenosis*) tested the diagnostic performance of online (in the catheterization laboratory) QFR computation for the diagnosis of hemodynamically significant coronary stenosis defined by FFR ≤0.80, in a larger cohort of patients (5 centers, 308 patients, 332 vessels) (5). Online assessment of QFR showed good correlation and agreement with FFR (*r* = 0.86; *p* < 0.001, and mean difference: 0.01 ± 0.06; *p* = 0.006, respectively). Interestingly, QFR was also useful in the evaluation of serial lesions, showing no difference between QFR and FFR in vessels with and without tandem lesions (−0.016 ± 0.068 vs. −0.004 ± 0.058; *p* = 0.10). Overall per-vessel diagnostic accuracy of QRF (the primary endpoint of the trial) was 92.7% (95% CI: 89.3% to 95.3%), which was significantly higher than the pre-specified value (*p* < 0.001). When compared to QCA, QFR demonstrated better discrimination performance (AUC 0.96 vs. 0.66; *p* < 0.001). Finally, this trial provided the information on additional time needed to undertake the QFR assessment (3D QCA reconstruction and frame count analysis) which took an additional 4.36 ± 2.55 min.

In parallel with FAVOR II China, The FAVOR II Europe-Japan Study was conducted (6). It was a prospective, observational study, which enrolled 329 patients enrolled in 11 international centers. The primary goal of the trial was to compare QFR sensitivity and specificity with 2D-QCA in order to detect significant lesions as defined by FFR criteria. This trial confirmed the possibility to perform QFR measurement in a catheterization laboratory in a time interval that was significantly shorter than the time needed to measure FFR (median time to QFR 5.0 min vs. median time to FFR 7.0 min, *p* < 0.001). QFR showed good per-vessel correlation and agreement with FFR (*r* = 0.83; *p* < 0.001, and mean difference, 0.01 ± 0.06, respectively) and larger AUC for QFR in comparison to 2D-QCA with FFR as a gold standard (0.92 [95% CI, 0.89–0.95] vs. 0.64 [95% CI, 0.57–0.70]; *p* < 0.001). Furthermore, there was no statistical difference between online and core-lab-determined QFR (rho 0.83; *p* < 0.001; mean difference, −0.03 ± 0.07). Finally, this trial suggested that in diabetic patients there could be some discrepancy between QFR and FFR values.

The WiFi II (*Wire-Free Functional Imaging II*) (7) was a Danish, two-center, prospective trial, which included 362 consecutive patients with suspected coronary artery disease on coronary computed tomographic angiography. All patients were sent to invasive coronary angiography. However, only 172 patients (with 255 lesions) were included in the final analysis; the majority of patients were excluded due to non-obstructive coronary artery disease (24%) and tight lesions (14%). QFR analysis was successful in 240 of 255 (95%) lesions. In this all-comers trial, QFR showed good correlation and precision compared to FFR (*r* = 0.70, *p* < 0.0001; mean difference of 0.01 ± 0.08, *p* = 0.08). In a subset of lesions chosen for repeatability analysis (the last 40 lesions analyzed by the same observer 6 months after the last QFR computation), there was excellent intraobserver variability with a mean QFR difference of 0.00 ± 0.06 (*p* = 0.65). The authors suggested an interesting QFR-FFR hybrid approach (with the QFR grey zone between 0.78 and 0.87) where FFR could be used for definitive decision-making. They estimated that a QFR-based approach (wire-free and adenosine-free procedures) could be performed in 68% of cases. This approach remains to be further validated.

In a recent study, Choi et al. (8) tested both the diagnostic performance of QFR and prognostic implications of QFR in terms of vessel-related composite outcomes at 2 years. Primary outcome consisted of composite of cardiac death, target-vessel myocardial infarction, and ischemia-driven target lesion revascularization. This was a Korean multicenter observational registry of patients who underwent invasive coronary angiography and FFR measurements. Like in previous trials, QFR was found to have excellent correlation and agreement with FFR (*r* = 0.860, *p* < 0.001; mean difference 0.002, 95% confidence limits −0.125 to 0.130). QFR was calculated offline and consistently showed high diagnostic performance regardless of vessel location, lesion length, or various clinical presentations including non-culprit vessels in acute coronary syndrome, previous myocardial infarction, diabetes mellitus, or gender. These findings support the generalizability of QFR evaluation in various clinical presentations, patient characteristics, or anatomical features. Finally, QFR showed significant predictive value for the occurrence of 2-year vessel-related composite outcomes. Vessels with QFR ≤0.80 had a significantly higher risk for these adverse events than vessels with QFR >0.80 (4.2% vs. 0.9%, HR 4.650, 95% CI, 1.254 to 17.240, *p* = 0.022).

### vFFR

The Fast Assessment of STenosis severity (FAST) study was the first study evaluating vessel fractional flow reserve (vFFR) based on 3D-QCA, both *in vitro* experimental model and in patients with stable angina or NSTEMI undergoing FFR evaluation (10). Three-dimensional reconstruction of the coronary artery was made using a total of three angiographic images (two separated at least 30° and a third to confirm the position of the sensor of the pressure wire). Computation of the pressure drop and vFFR was performed offline using computer simulation of blood flow. Of importance, the value of the aortic pressure of the specific patient measured in the catheterization laboratory was used for this computation, while the maximum hyperaemic blood flow was empirically determined from clinical data. Clinical data demonstrated a good correlation (*r* = 0.89; *p* < 001) between FFR and vFR, as well as a good agreement between them (mean difference = 0.01, SD = 0.0356). Furthermore, the correlation between FFR and vFR was good in both stable and NSTE-ACS patients (*r* = 0.89 vs. 0.89, respectively), while vFR had a high accuracy to detect FFR ≤0.80 [AUC 0.93 (95% CI: 0.88–0.97)], which is a standard threshold for performing PCI.

Because of the relatively small sample size of the FAST trial (100 patients), no conclusions could be made about the performance of vFFR in different lesion subsets. Therefore, a larger FAST EXTEND trial was conducted to evaluate its performance in complex lesions (final population of 294 patients with stable angina or NSTE-ACS) (11). This was a retrospective cohort study with offline assessment of vFFR, which excluded about 60% of cases initially selected for the trial because of not meeting inclusion criteria. Still, it confirmed excellent diagnostic performance and a strong correlation between vFFR and FFR in different coronary vessels and lesion subsets (bifurcations, tortuous vessels, calcified lesions, tandem lesions, and diffuse disease).

To overcome previous limitations, the FAST II (*Fast Assessment of STenosis severity*) study was conducted (12). It was a prospective trial involving six centers, which enrolled 334 patients (with stable angina or NSTE-ACS), aimed to evaluate the performance of vFFR to FFR as a reference standard in a prospective manner. Computation of vFFR was performed offline by a blinded core laboratory, and also assessed on-site. This trial confirmed a good correlation of both core-lab and on-site vFFR with FFR as reference (*r* = 0.74, *p* < 0.001 and *r* = 0.76, *p* < 0.00), with small absolute differences (0.0029 ± 0.0642 and 0.0057 ± 0.0666, respectively). Furthermore, vFFR showed a good correlation with FFR in various lesion and patient subsets (diffuse disease, focal disease, bifurcations, calcified lesions, diabetic patients), which could support the use of this index in routine clinical practice.

### FFRangio

FFRangio is primarily based on a 3-dimensional reconstruction of the whole coronary tree followed by computer-based rapid flow analysis, allowing calculation of FFRangio from routine angiograms within a few minutes of automatic processing. For 3D reconstruction of the coronary tree at least 2 different angiographic projections are needed, recorded at 15 frames/s, with special care taken to fill the arteries completely as possible with angiographic contrast. The exact angiographic projections and separations are left to the operator's discretion (13).

In the pilot study for FFRangio, a total of 101 lesions in 88 patients were analyzed and compared to pressure wire-based FFR measurements. This was first-in-man study of FFRangio which indicated high reproducibility and diagnostic accuracy of FFRangio compared to invasive FFR (*r*^2 ^= 0.868) (13).

In order to further evaluate the diagnostic accuracy and interobserver reproducibility of FFRangio compared to invasive FFR, another trial was conducted (14). A total of 199 patients were enrolled at four interventional centers, while 184 patients with 203 coronary lesions were included in the final analysis. Calculation of FFRangio required 2 to 3 conventional radiographic projections, in which the lesions can be clearly traced, with images in high resolution (≥700 × 700) and a frame rate of at least 10 frames/s. Excellent interobserver reproducibility was demonstrated (intraclass correlation coefficient was 0.962), with good agreement to invasive FFR measurements [*r* = 0.88, with small estimated bias of 0.007 (95% CI: −0.096 to 0.112) that does not systematically underestimate or overestimate invasive FFR values]. To provide the blinding of the operators, these analyses were performed offline. Therefore, it was not possible to estimate how much these additional steps could prolong the invasive procedure (14).

Kornowski et al. demonstrated that FFRangio could be used online during coronary angiography (15). In a small trial including 53 patients and 60 lesions, they showed good correlation (*r* = 0.91; *p* < .001) and agreement (95% limits of agreement from −0.065 to 0.07 using Bland-Altman analysis) with wire-based FFR. Additional time needed to calculate FFRangio after completion of the diagnostic angiography was approximately 10 min (15).

The main limitation of previous trials was a relatively small number of patients and centers, with diagnostic angiograms performed by only a few operators. To overcome these issues, the FAST-FFR trial (*FFRangio Accuracy versus Standard FFR*) was conducted. It included 301 patients (319 vessels) at 10 centers in USA, Europe, and Israel. The 3D reconstruction of the coronary tree was taken from 2 or more projections (with a minimum separation of 30°) taken at 10 frames/s at least. The average processing time for FFRangio on-site calculation was only 2.7 min (not including the time needed for correction of the coronary reconstruction and lesion identification). Both sensitivity and specificity of FFRangio for predicting FFR were high and both exceeded the prespecified goals (93.5% and 91.2%, respectively). Of importance, the sensitivity, specificity, and diagnostic accuracy of FFRangio remained high even when FFR values were near the cut-off between 0.75 and 0.85 (89%, 85%, and 87%, respectively). This study further confirmed the strong correlation between FFR and FFRangio (*r* = 0.80, *p* < 0.001). Authors suggest that FFRangio has a unique feature that discriminates it from both invasive FFR and other commercially available non-invasive tools, being the 3D reconstruction of the complete coronary tree with FFR values measured along each vessel. This may ease the analysis of coronary stenosis and may influence the choice of revascularization strategy, which should be further investigated (16).

Previously discussed trials tested the performance of FFRangio mainly in single-vessel disease patients. To evaluate FFRangio to reference standard FFR in multivessel disease, a single-center Japanese trial was conducted (17). This study enrolled 50 patients with 118 lesions, while 36% had triple vessel disease. The authors were able to demonstrate the excellent diagnostic performance of FFRangio compared to FFR with per lesion sensitivity of 92.3% (95% CI 79.1%–98.4%), specificity 92.4% (95% CI 84.3%–97.2%), and diagnostic accuracy 92.4% (95% CI 87.4%–97.3%). Of interest, the mean time required for measuring FFR was 15.9 ± 8.9 min per patient (6.9 ± 5.6 min per lesion), compared to 9.6 ± 3.4 (4.3 ± 3.4 min per lesion) for FFRangio (*p* < 0.001) (although the FFRangio analysis was conducted offline) (17).

Furthermore, a pooled analysis of all 5 previously mentioned prospective cohort studies on FFRangio was published, including 700 lesions in 588 patients (18). FFRangio showed powerful and consistent diagnostic performance across different patient and lesion subgroups; sensitivity, specificity and diagnostic accuracy for FFRangio (compared to the binary cutoff for FFR of 0.80) were 91%, 94%, and 93%, respectively. There was excellent correlation between FFR and FFRangio [0.83 (*p* < 0.001)], with the C-statistic for FFRangio of 0.95 (*p* < 0.001), and the minimum difference between FFR and FFRangio (0.00 ± 0.12) (18).

Finally, in *post hoc* analysis of the FAST-FFR trial, FFRangio was shown to have high diagnostic performance regardless of the patient (age, gender, clinical presentation, body mass index, and diabetes) and most lesion characteristics (calcification, tortuosity, and lesion location). Interestingly, sensitivity was equally high across all coronary vessels, while specificity was highest in the LAD and lower in the RCA and circumflex arteries (about 85%, *p* < 0.05), which will need confirmation in further trials (25).

### caFFR

The FLASH FFR study was the first clinical trial aimed to compare computational pressure-flow dynamics derived FFR (caFFR), applied to coronary angiography, with invasive FFR. It was a prospective, multicenter trial conducted at six centers in China. Measurements were performed in 328 patients/coronary arteries, with only one lesion evaluated per patient. For the calculation of caFFR, aortic pressure was simultaneously recorded during angiography using a specialized pressure transducer (*FlashPressure, Rainmed Ltd, Suzhou, China*). This approach of using real-time invasive pressure coupled with computational flow modeling used for caFFR calculation could possibly provide an advantage over other angiography-derived FFR indices. On average, the total operating time for caFFR measurement was 4.54 ± 1.48 min. The reported diagnostic accuracy of online caFFR was 95.7%, with a high correlation between caFFR and FFR (caFFR = 0.78*FFR −0.18, *R* = 0.89), and no systematic difference between two indices (mean difference of −0.002 ± 0.049). Furthermore, caFFR performed well even in the “grey zone” of FFR and/or diameter stenosis. The caFFR diagnostic accuracy was 89.9% in 119 vessels with FFR between 0.75%–0.85%, and 95.6% in 294 vessels with diameter stenosis 40%–80% (20).

In one smaller trial with 104 patients, caFFR was compared to invasive FFR before (104 patients) and after (65 patients) PCI. The authors reported a good correlation between caFFR and FFR measurements before (*r* = 0.77; *p* < 0.001) and after (*r* = 0.82; *p* < 0.001) stent implantation (21). Although they reported some adverse clinical outcomes to be related to low post-procedural caFFR, the number of events and participants is too low to draw such a conclusion, which should be addressed in larger prospective trials.

### AccuFFRAngio

Recently, another angiography-based index was proposed named AccuFFRAngio. It uses three-dimensional QCA obtained from two angiographic images separated for at least 25 degrees, and TIMI frame count applying computational fluid dynamics theory to calculate it. In the first reported clinical trial, which included 300 patients with stable angina, a good correlation between AccuFFRAngio and FFR was observed (*r* = 0.83, *p* < 0.001). Furthermore, there were good agreements between AccuFFRangio and FFR with a mean difference value of −0.001 (limits of agreement: −0.124 to 0.122) when aortic pressure was measured at the coronary ostium and −0.030 (limits of agreement: −0.155 to 0.095) when it was set to 100 mmHg. Overall diagnostic accuracy for AccuFFRangio was 93.7% (22).

These results were further confirmed in another trial performed in 298 patients/coronary arteries (23). This trial showed excellent correlation between computed AccuFFRangio and measured FFR (*r* = 0.812, *p* < 0.001), with the AccuFFRangio AUC value of 0.96.

## Influence of lesion subsets and clinical scenarios on diagnostic performance of angiography-derived FFR

Initial validation studies excluded patients with challenging lesion anatomies like ostial lesions (due to lack of proximal reference segment), left main stenosis, and bifurcations. Also, patients with complex clinical features were also initially excluded (acute myocardial infarction, microvascular dysfunction, heart failure, aortic stenosis). As angiography-derived FFR use was expanding, more data became available on some of these lesion/patient subsets.

Assessment of severity of left main coronary stenosis remains challenging, and frequently requires combination of angiographic, functional, and assessment using intracoronary imaging. There is limited but encouraging experience with angiography-derived FFR (mainly QFR and vFFR) in evaluation of the left main, with good correlation with both intravascular imaging and physiological assessment (26–30).

In an all-comer observational registry (*Catholic Imaging and Functional Research (C-iFR) Cohort*) with total of 1,012 patient (1,265 vessels), diagnostic accuracy of QFR (compared to FFR) in the group with bifurcation lesions was not-significantly lower than that of non-bifurcation lesions (97.26% vs. 94.79%, *p* = 0.069) (31). Majority of angiography-derived software solutions reconstruct main vessel only, and do not take into account the side branch stenosis and flow, which makes bifurcation assessment challenging.

Coronary artery disease is common in patients with severe aortic stenosis (AS), and functional assessment of lesion severity could be challenging. Aortic stenosis leads to LV pressure overload, causing ventricular remodeling and hypertrophy. Increased LV mass and intracavity pressure induces changes in physiology which could impact the assessment of ischemia using both FFR and iFR. It is suggested that iFR may represent a better option in patients with AS since it does not require adenosine and is independent of systolic flow (32). There is growing evidence that there is possible role of angiography-derived FFR in patients with AS. Kleczynski et al. demonstrated that in patients with AS, the QFR had good agreement with both FFR and iFR, while the agreement appears to be even better when the iFR is used as the reference (33). In a small trial in 28 patients undergoing TAVI for the treatment of AS, pre-TAVI QFR had a higher classification agreement with post-TAVI FFR compared to pre-TAVI FFR, which could suggest that QFR could be less influenced by AS in the evaluation of coronary artery disease (34). However, the role of angiography-derived FFR in AS needs to be further addressed in larger prospective clinical trials.

## Clinical outcomes of PCI guided by fractional flow reserve derived from invasive coronary angiography

In order to be adopted in clinical practice, these non-invasive angiography-derived FFR indices must be proven to provide clinicians with information that is at least non-inferior to wire-based indices in prognostic terms. If that would be achieved, these techniques would overcome many limitations of the wire-based approach, with a huge potential to widen the physiological assessment of coronary lesions in everyday patients. Currently, only a few clinical trials providing us with this information were published, while many are ongoing. The actual chapter aims to review these trials.

### QFR

All previously discussed indices have good diagnostic performance as compared to wire-based FFR, which may direct their practical use. Nevertheless, the ultimate goal is to demonstrate that patients whose treatment is based on these parameters could have non-inferior (or superior) clinical outcomes in comparison to FFR-guided PCIs. Currently, only a few trials have been published testing clinical outcomes, while a few others are ongoing.

FAVOR III China was a multicentre (performed at 26 hospitals in China), randomized clinical trial (NCT03656848). It enrolled 3,825 patients that were randomly assigned into two groups. QFR-guided strategy was performed in one group (where PCI performed only if QFR ≤0.80), while the other group had angiography-guided strategy, with standard visual angiographic assessment of coronary lesions. The primary endpoint was the 1-year rate of major adverse cardiac events, defined as a composite of death from any cause, myocardial infarction, or ischemia-driven revascularisation. The composite primary endpoint occurred within 1 year in 110 (5.8%) of 1,913 patients in the QFR-guided group and in 167 (8.8%) of 1,912 patients in the angiography-guided group (difference −3.0% [95% CI −4.7 to −1.4; HR 0·65 [95% CI 0.51 to 0.83]; *p* = 0·0004). These results suggest that QFR-guided PCI could improve 1-year clinical outcomes compared with standard angiography guidance (9). Of importance, the operators were not blinded to treatment allocation, and patients with complex anatomies were excluded from the trial.

More data on the potential role of QFR-guided PCI comes from a recently published *post hoc* analysis of a randomized PANDA III trial. QFR was retrospectively calculated from angiograms of 1,391 patients, who were divided into two groups: QFR-consistent treatment (i.e., patients in whom all functionally ischaemic vessels (baseline QFR ≤0.80) were treated and in whom all non-ischaemic vessels (baseline QFR >0.80) were deferred) and QFR-inconsistent treatment (if previous criteria were not fulfilled). The authors reported that 58.5% of patients were treated in accordance with what the QFR measurement would have recommended. Even after statistical adjustment for baseline differences between groups, 2-year rates of MACE remained significantly lower in the QFR-consistent group (8.8% vs. 13.6%; adjusted HR 0.64, 95% CI: 0.44–0.93), mainly due to reduced ischemia-driven revascularisation (2.9% vs. 8.0%; adjusted HR 0.35, 95% CI: 0.20–0.60) (35).

The efficacy of QFR-guided PCI is currently being tested in three prospective, randomized trials. One of them if the FAVOR III Europe Japan Study (FAVOR III EJ; NCT03729739) which will recruit 2,000 patients. This study will investigate whether QFR-based diagnostic strategy is non-inferior to a standard pressure-wire guided strategy (FFR) in the evaluation of patients with stable angina and intermediate coronary stenosis. Non-inferiority will be clinically determined after the 1-year follow-up. The estimated study completion date is December 2025.

Currently, there is a paucity of data regarding the best functional parameter(s) for the evaluation of coronary artery disease in patients with significant valvular disease who are planning for valvular surgery. The FAVOR4-QVAS (NCT03977129) trial will try to address this important clinical issue. This study will enroll 792 patients undergoing elective open-heart valvular surgery due to primary valvular heart disease, who also have visually estimated stenotic coronary lesions of ≥50%. The FAVOR4-QVAS study will test whether QFR-guided strategy can reduce the MACE risk within 30 days after surgery, compared with the coronary angiography guided strategy. Except for ischemic endpoints (all-cause death, myocardial infarction, stroke, and unplanned coronary revascularization), MACE will also include new renal failure requiring dialysis. The estimated study completion date is December 2026.

Another trial currently recruiting patients is PIONEER-IV (NCT04923191). A total of 2,540 patients will be randomized to either angio-based physiology guidance (QFR) or local routine diagnostic procedure (LRDP) and usual care in an all-comers patient population (including patients with high bleeding risk) undergoing PCI with unrestrictive use of the HT Supreme sirolimus-eluting stent. The estimated study completion date is January 2026.

### vFFR

The FAST III (NCT04931771) is a randomized controlled, open-label, multicenter (approximately 35 sites in 7 European countries), non-inferiority trial. A total of 2,228 participants will be randomized to vFFR- or FFR-guided revascularization. The primary endpoint (composite of all-cause death, any myocardial infarction, or any revascularization) will be analyzed 12 months after randomization. The estimated study completion date is May 2024.

The Comparison of vessel-FFR versus FFR in intermediate coronary stenoses trial (LIPSIASTRATEGY trial, NCT03497637) is a prospective, randomized, controlled, multicenter, open-label study. This non-inferiority trial will compare vFFR to FFR in the assessment of intermediate coronary stenosis. It is estimated to enroll 1,926 patients and to assess the occurrence of primary end-point (MACE—a composite of cardiac death, nonfatal myocardial infarction, or unplanned revascularization) during 12 months after randomization. It is planned to be completed by November 2026.

### FFRangio

Outcomes of PCI guided by FFRangio were tested in a cohort trial conducted in two centers from Israel and Japan that adopted this diagnostic tool for the treatment decisions in everyday patients, without the concomitant measurement of wire-based FFR. Although this was not a randomized comparison with FFR, these data suggest favorable outcomes in both revascularized and deferred patients. This trial included 492 patients with 552 coronary lesions where treatment decisions (to revascularize or to defer) were consistent with FFRangio results (revascularization for lesions with FFRangio ≤0.8 or deferral for lesions with FFRangio >0.8). At 1 year, MACE (the composite of cardiovascular mortality, myocardial infarction, and repeat revascularization) occurred in 6 and 9 patients in the deferral and revascularization groups (2.5% and 4.1%), respectively. The authors concluded that these event rates were relatively low and comparable to previously reported outcomes of FFR-guided PCI (19, 36).

Recently, another registry-based trial has been designed and will assess clinical outcomes of FFRangio-based decisions (deferral or revascularization). The Japan FFRangio Clinical Outcomes Study (NCT05648396) will recruit 2,500 real-world patients in Japan and Israel through a retrospective multicentre registry. The primary endpoint will be a composite of all-cause death, myocardial infarction, and repeat revascularization at 1-year follow-up. It is expected to be completed by 2027.

### caFFR

Currently, there are no published results regarding the outcomes of the caFFR-based strategy of myocardial revascularization. The ongoing FLASH II trial (NCT04575207) will investigate whether caFFR, compared with FFR measured by a pressure wire, has a non-inferior clinical effect and cost-benefit in guiding the PCI in patients with moderate coronary artery stenosis in terms of long-term clinical prognosis. It will enroll 2,132 patients who will be followed for 2 years. The primary endpoint will be MACE which will comprise all-cause death, myocardial infarction, and unplanned revascularization. It is estimated to be completed by June 2024.

### AccuFFRangio

To our best knowledge, there are no published data regarding revascularization based on AccuFFRangio, while two trials are ongoing. The Prognostic Implications of AccuFFRangio-based functional evaluation for guiding coronary intervention for non-IRA stenosis in patients with STEMI trial (NCT05209503) is a prospective, single-center (*Wuhan Asia Heart Hospital, China*) clinical trial that will compare AccuFFRangio- and angiography-guided PCI of non-infarct related arteries after dilatation of culprit artery in patients with ST-elevation myocardial infarction. It will enroll 500 patients which will be followed for 1 year. The primary endpoint will be vessel-oriented composite endpoints (VOCEs: composite of vessel-related cardiovascular death, vessel-related myocardial infarction, and ischemia-driven target vessel revascularization). The trial will also search for optimal values of post-PCI AccuFFRangio values that are related to favorable outcomes. The Diagnostic performance and prognostic ability of AccuFFRangio for non-IRA in NSTE-ACS patients is the trial conducted by the same study group, with almost the same design as the previous one, but in NSTE ACS patients (NCT05202041). Both are planned to be completed in 2023.

## Use of angiography-derived FFR to optimize the PCI procedure

Beside selection of lesions that should be treated with PCI (by measuring FFR before PCI), wire-based post-PCI FFR could provide us important information regarding residual disease, suboptimal stent implantation, and long-term clinical outcomes (37–39). There is growing evidence that angiography-derived FFR after PCI could give us similar information.

In a retrospective analysis of SYNTAX II trial, post-PCI QFR was successfully measured in 771 of total 968 vessels (analyzability of 79.6%). The authors demonstrated that low post-PCI QFR (<0.91) was related to worse clinical outcomes (vessel-oriented composite endpoint (VOCE) at 2 years: a composite of vessel-related cardiac death, vessel related myocardial infarction, and target vessel revascularization) as compared to values ≥0.91. The post-PCI QFR cutoff of 0.91 for the prediction of 2-year VOCE had sensitivity of 65% and specificity of 64% (AUC: 0.702; 95% CI: 0.633 to 0.772; *p* < 0.001). Authors identified the following factors that were associated with lower post-PCI QFR values: previous myocardial infarction, serial lesions, LAD stenosis, lower pre-procedural QFR value, and lower minimal surface area derived from IVUS (40).

In the international multicenter prospective HAWKEYE Study, vessels with vessel-oriented composite endpoint during follow-up had lower values of post PCI QFR as compared to vessels without adverse outcomes (0.88 vs. 0.97, *p* < 0.001). ROC analysis defined best cutoff of post PCI QFR ≤ 0.89 (AUC 0.77; 95% confidence interval: 0.74–0.80; *p* < 0.001). Furthermore, it was estimated that post PCI QRF ≤ 0.89 was related to 3-fold increase in risk for the vessel-oriented adverse outcomes (41).

In a FAST POST clinical trial, one hundred patients with dedicated microcatheter-based measurements of FFR after PCI and 2 adequate angiographic views with calculated post-PCI vFFR were included in analysis. Post-PCI FFR had good agreement (mean values of 0.91 ± 0.07 and 0.91 ± 0.06, respectively) and good correlation (*r* = 0.88; *p* < .001) with vFFR (42).

The FAST Outcome study assessed the prognostic value of post-PCI vFFR on the incidence of target vessel failure (TVF—a composite endpoint of cardiac death, target vessel myocardial infarction and target vessel revascularization (TVR) at 5-year follow up) in 748 patients (832 vessels) with available orthogonal angiographic projections of the stented segment. They demonstrated that lower post-PCI vFFR values were associated with a significantly increased risk of TVF and TVR at 5-years follow-up. Vessels in the lowest tertile of vFFR (<0.88) had a 1.8 fold increase in the risk of TVF at 5-years follow-up (43).

The post-PCI coronary angiography-derived FFR (caFFR) was tested in retrospective analysis in 136 patients with 159 vessels treated with PCI. The mean post-PCI caFFR was 0.90 ± 0.06, while the median trans-stent caFFR gradient was 0.04 (interquartile range 0.02–008). Although low number of adverse events occured, the TVF rate was significantly higher in patients with post-PCI caFFR < 0.90 (4 [8.16%] vs. 1 [1.15%], *p* = 0.037). They also reported that suboptimal post-PCI caFFR and trans-stent caFFR gradient were common immediately after stenting (44).

## Detection of microcirculatory disfunction using angiography-derived parameters

Microcirculatory disfunction assessment is currently limited to measurement of invasive indices such as index of microcirculatory resistance (IMR, using a pressure-temperature sensor guidewire) and hyperemic microvascular resistance (HMR, using a pressure-flow velocity sensor guidewire), both of which require complex intracoronary measurements. There are novel approaches for the assessment of IMR that are angiography-based (applying the same methodology of QFR or caFFR computation) which are in the early phase of development. Initial results look promising, showing good correlation with invasively measured IMR (45–48), leading to the possibility that this approach could give physicians some important insight into the microcirculatory properties downstream the interrogated coronary artery.

## Limitations of fractional flow reserve derived from invasive coronary angiography

The feasibility of measuring FFR derived from coronary angiography in real clinical practice, in an all-comers population, remains controversial. Although the feasibility was usually high in prospectively collected data in clinical trials, retrospective series of patients showed a lower feasibility rate. Thus, in retrospective analysis from the SYNTAX II trial, QFR was analyzable in 71.0% of lesions, while per-patient-level QFR analysis of all angiographic stenoses in the 3 vessels was feasible in only 28.2% of patients. QFR was not analyzable mainly because of the absence of 2 appropriate projections (severe vessel overlaps or tortuosity at the lesion, etc.) (49). Furthermore, in 1,003 patients referred to the heart team for discussion, in 440 patients (43.9%), there was a screening failure due to insufficient quality of the coronary angiogram, which did not allow calculation of vFFR (50). Having in mind these data, it is important to take special care in obtaining high-quality angiographic images in at least two projections of every lesion, especially if we plan to retrospectively (later) measure FFR derived from angiographic images. Online measurements, while the patient is still in the cath lab, usually do not have such a limitation. Finally, the potential role of QFR in heart team decisions is being investigated in ongoing DECISION QFR randomized trial (51).

All FFR parameters that are the subject of this review differ in whether they use only 3D anatomic reconstruction of the coronary arteries or add some patient-specific physiological input like aortic pressure or TIMI frame count. It remains unknown whether the addition of these physiological data could provide some important advantages of those indices. Direct head-to-head comparisons are lacking and warrant further investigation.

Correct calculation of angiography-derived FFR critically depends on the operator's experience. It is demonstrated that when an expert reanalysis previous 3D reconstruction of casual users, it may result in a change in the treatment decision in as much as 37% of cases (52).

Of all FFR indices discussed in our review, only FFRangio uses a 3D reconstruction of the whole coronary tree, while others reconstruct only the main vessel. It remains unknown how calculated values are influenced by large side branches, especially in situations when the interrogated vessel is a donor of collaterals or when it receives collateral flow (either from a native artery or coronary bypass graft).

Angiography-based FFR calculation is an emerging and promising alternative to well-established pressure-wire measured FFR, with the potential to extend the utilization of coronary physiology guidance of PCI. It is the topic of intensive scientific research, and future data, especially on clinical outcomes, will define its role in real clinical practice.

## Summary

Angiography-derived indices for the evaluation of coronary circulation have the potential to improve clinical practice and bring physiologically-based decisions on revascularization to more patients in catheterization laboratory. Advances in computational methods and integration of these solutions in real-time angiography systems will reduce the need for invasive measurements, reduce the cost and time of the procedure, and eventually bring the prognostic benefit to the patients. Intensive research in this area with different competitors and approaches will hopefully give us many expected answers regarding its clinical applicability.
